# Pacemaker lead-associated tricuspid regurgitation in patients with or without pre-existing right ventricular dilatation

**DOI:** 10.1007/s00392-021-01812-3

**Published:** 2021-02-10

**Authors:** Martin Riesenhuber, Andreas Spannbauer, Marianne Gwechenberger, Thomas Pezawas, Christoph Schukro, Günter Stix, Matthias Schneider, Georg Goliasch, Anahit Anvari, Thomas Wrba, Cesar Khazen, Martin Andreas, Günther Laufer, Christian Hengstenberg, Mariann Gyongyosi

**Affiliations:** 1 Department of Cardiology, Medical University of Vienna, Vienna, Austria; 2 Medical University of Vienna, IT Systems and Communications, Vienna, Austria; 3 Department of Cardiac Surgery, Medical University of Vienna, Vienna, Austria; 4 Department of Internal Medicine II, Division of Cardiology, Medical University of Vienna, Währinger Gürtel 18-20, 1090 Vienna, Austria

**Keywords:** Tricuspid regurgitation, Pacemaker, Right ventricle, Valvular heart disease, Device complications

## Abstract

**Background:**

Transcatheter tricuspid valve intervention became an option for pacemaker lead-associated tricuspid regurgitation. This study investigated the progression of tricuspid regurgitation (TR) in patients with or without pre-existing right ventricular dilatation (RVD) undergoing pacemaker implantation.

**Methods:**

Patients were included if they had implantation of transtricuspid pacemaker lead and completed echocardiography before and after implantation. The cohort was divided in patients with and without RVD (cut-off basal RV diameter ≥ 42 mm). TR was graded in none/mild, moderate, and severe. Worsening of one grade was defined as progression. Survival analyses were plotted for 10 years.

**Results:**

In total, 990 patients were analyzed (24.5% with RVD). Progression of TR occurred in 46.1% of patients with RVD and in 25.6% of patients without RVD (*P* < 0.001). Predictors for TR progression were RV dilatation (OR 2.04; 95% CI 1.27–3.29; *P* = 0.003), pre-existing TR (OR 4.30; 95% CI 2.51–7.38; *P* < 0.001), female sex (OR 1.68; 95% CI 1.16–2.43; *P* = 0.006), single RV lead (OR 1.67; 95% CI 1.09–2.56; *P* = 0.018), mitral regurgitation (OR 2.08; 95% CI 1.42–3.05; *P* < 0.001), and enlarged left atrium (OR 1.98; 95% CI 1.07–3.67; *P* = 0.03). Survival-predictors were pacemaker lead-associated TR (HR 1.38; 95% CI 1.04–1.84; *P* = 0.028), mitral regurgitation (HR 1.34; 95% CI 1.02–1.77; *P* = 0.034), heart failure (HR 1.75; 95% CI 1.31–2.33; *P* < 0.001), kidney disease (HR 1.62; 95% CI 1.25–2.11; *P* < 0.001), and age ≥ 80 years (HR 2.84; 95% CI 2.17–3.71; *P* < 0.001).

**Conclusions:**

Patients with RVD receiving pacemaker suffered from increased TR progression, leading to decreased survival.

**Graphic abstract:**

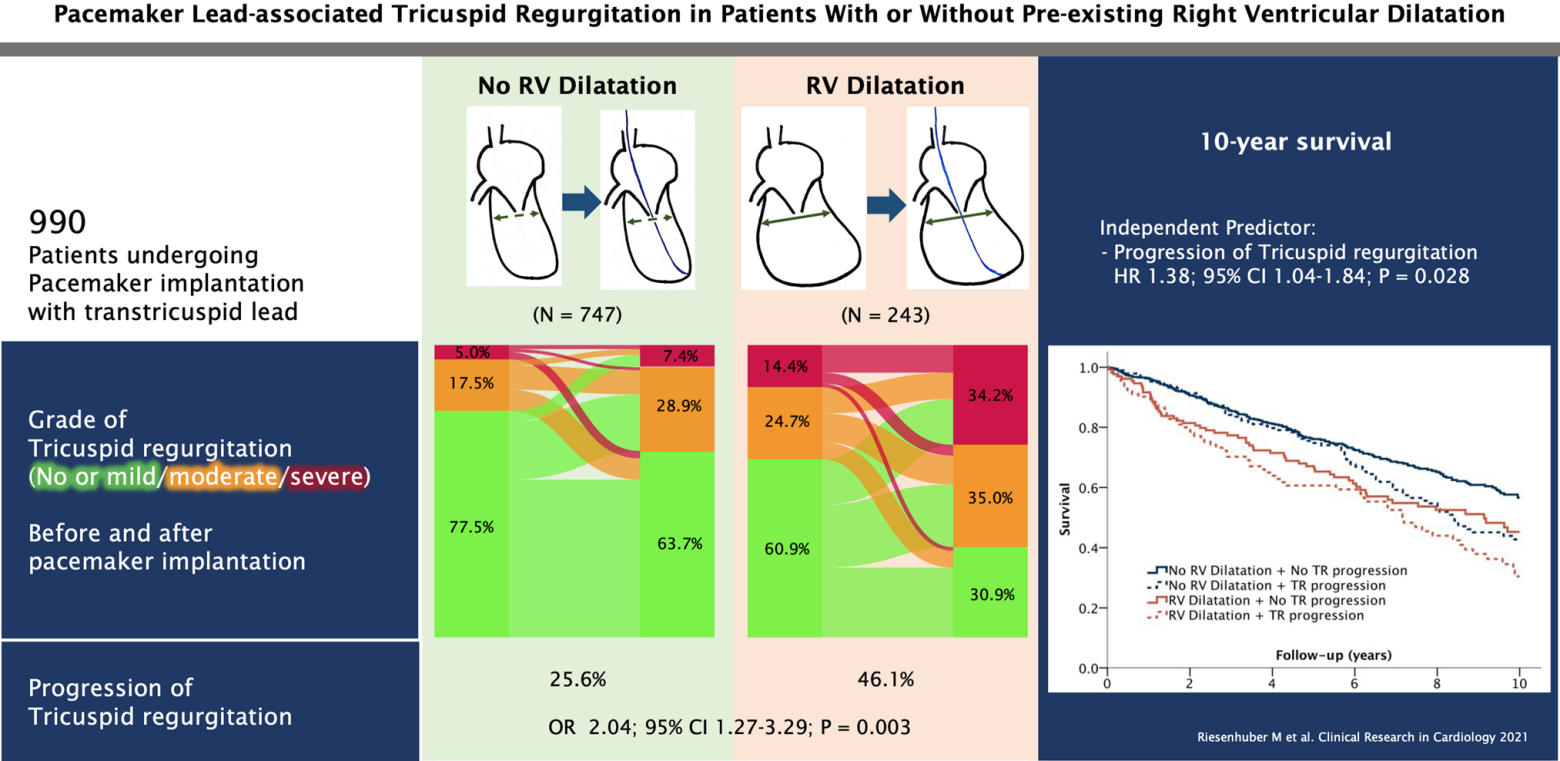

## Introduction

Tricuspid regurgitation (TR) represents a major burden of cardiovascular disease, and the prevalence of TR is comparable to the prevalence of aortic stenosis [1]. Approximately three million patients in Europe and 1.6 million patients in the United States suffer from clinically relevant TR [2]. TR is an independent risk factor for increased mortality [3], and surgical treatment of TR is associated with significant perioperative mortality [4, 5]. Therefore, concepts for efficient percutaneous interventional treatments of TR are on the rise. Transcatheter tricuspid valve interventions (TTVI) represent a safe, effective, and alternative treatment approach for secondary TR [6, 7], and improve the severity of secondary TR, symptoms, and hospitalizations for heart failure [8–10]. However, TTVI is offered only in tertiary centers and is not yet standard of care.

Interference of a right ventricular transtricuspid pacing lead with the tricuspid valve might contribute to or cause TR, and is reported in 7% to 45% of RV lead implantations [11]. RV leads of cardiac implantable electronic devices (CIEDs) are associated with progression of TR, which is associated with poor outcomes [12–17]. Lead-associated TR affects long-term RV function, which is linked to decreased survival [18].

CIED-induced TR can be divided in primary and secondary CIED-induced TR, and recent data found up to 60% of worsened TR after CIED implantation are of secondary origin [19]. While primary CIED-induced TR is caused by direct interaction of the lead and the tricuspid valve, secondary CIED-induced TR has its origin in RV dilatation due to pacing/heart failure. Untreated primary CIED-induced TR triggers RV dilatation due to volume overload, and leads to secondary TR. If this “point of no return” is reached, lead extraction could not reverse TR [19, 20].

Currently, only one representative study is published addressing TTVI in patients with CIEDs: Taramasso et al. analyzed 470 patients with severe TR undergoing TTVI, and compared patients with and without CIED [21]. Equal treatment outcomes could be demonstrated in terms of rates of procedural success, residual TR, symptomatic improvement, and survival.

In the era of increasing TTVI, CIED-induced TR has to be critically re-evaluated with special attention for primary and secondary CIED-induced TR. Considering predictable CIED-induced TR, leadless pacing could become of interest. If secondary CIED-induced TR occurs, TTVI could be an effective treatment strategy.

The aim of this study was to evaluate pacemaker lead-associated TR regarding right heart morphology (dilated vs. non-dilated RV) and identify predicting factors for pacemaker lead-associated TR and survival.

## Methods

### Study design

This retrospective cohort study is based on patients of the Department of Cardiology at the Medical University of Vienna, who had a first pacemaker implantation and echocardiographic studies 17 months (IQR 2–55) before and 5 months (IQR 0.1–21) after the implantation.

This study followed all principles of the Declaration of Helsinki and was approved by the Ethics Committee of the Medical University of Vienna (EK 1525/2015).

### Patients

All patients with implantation of a cardiac pacemaker with a transtricuspid pacing lead were included in the study, if they had an echocardiography before and after pacemaker implantation. Patients were enrolled from May 2000 to April 2015. Patients with implantable cardioverter defibrillator (ICD), cardiac resynchronization therapy (CRT) or single-chamber pacemakers with only an atrial pacing lead were excluded from the study.

### Baseline parameters

The comorbidities (coronary artery disease, heart failure, diabetes mellitus, myocarditis, “any” atherosclerosis, previous stroke or transient ischemic attack (TIA), previous coronary artery bypass grafting (CABG), atrial fibrillation, chronic kidney disease, endocarditis) were derived from the hospital information system based on ICD-10 codes. Patients with heart failure included those with reduced or preserved ejection fraction. The diagnosis of diabetes included patients with type 1 or type 2 diabetes.

### Clinical follow-up of the patients

Clinical follow-up up to 10 years was available in 562 patients, and 5569 person-years were analyzed. Mortality data (time of death) were obtained from the Federal Institute under Public Law “Statistics Austria”. Pacemaker lead replacements were defined by implantation of at least one new lead (atrial or ventricular) with or without lead extraction within the clinical follow-up.

### Echocardiography

Transthoracic echocardiography studies were performed before and after pacemaker implantation. All echocardiographic studies were performed by board-certified physicians, and high-end scanners (Vivid E9, Vivid7, GE Healthcare, Chicago/Illinois, USA) with 2.5-MHz transducers were used. The conducted 2-dimensional echo exams included parasternal, apical, and subcostal views with M-mode, 2-dimensional echocardiography, and conventional and color Doppler ultrasonography according to current recommendations [22, 23]. TR and mitral regurgitation were graded by visual estimation under consideration of an integrated approach including valve morphology, color flow jet, continuous wave signal of the jet, vena contracta width, and proximal isovelocity surface area (PISA) radius as recommended by the guidelines of the European Association of Echocardiography and the American Society of Echocardiography [24, 25], and were categorized in three groups: no or mild, moderate, and severe. Lead-associated progression of TR was defined by progressing of TR from no/mild to moderate or severe, or from moderate to severe comparing echocardiography studies before and after pacemaker implantation. RV was graded as dilated if the basal RV end-diastolic diameter was 42 mm or larger in 2D echocardiography (4-chamber view) [22, 23]. Further visual assessment of the RV was performed in parasternal long and short axis and subxiphoidal views. Right and left atria were graded as significantly dilated if the longitudinal diameter in the apical 4-chamber view exceeded 70.0 mm. Left ventricular function (LVF) was categorized as normal, mildly [left ventricular ejection fraction (LVEF) 41–53% in women and 41–51% in men], moderately (LVEF 30–40%) or severely (LVEF < 30%) impaired after visual estimation [22, 23].

Peak tricuspid regurgitation velocity (TR-*V*_max_) was assessed by continuous wave (CW) Doppler. Systolic pulmonary artery pressure (sPAP) was calculated as the sum of the tricuspid jet gradient (assessed by Doppler) and right atrial pressure. Right atrial pressure was estimated by visualizing the inferior vena cava and its response to respiration. Presence of significant PH was assumed if sPAP was ≥ 56 mmHg or TR-*V*_max_ was ≥ 3.5 m/s.

### Statistical analysis

Statistical analyses were performed with SPSS software (version 24.0; Macintosh; SPSS IBM). Continuous variables were tested for normal and non-normal distribution, and means ± standard deviations were calculated. Groups with continuous variables were compared with the t-test (normal distribution) or with the Mann–Whitney *U* test (non-normal distribution). Discrete variables were compared with the chi-squared test. Odds ratios for progression of TR were calculated with a logistic regression model. Predicting factors for survival (baseline echocardiographic and clinical parameters) were determined with COX regression with a follow-up of 10 years, and hazard ratio and 95% confidence intervals were reported. Parameters were included in the multifactorial regression models if the *P* value was < 0.10 in the univariate regression. A two-sided *P* value < 0.05 was considered statistically significant.

## Results

### Baseline patient parameters

Baseline characteristics of the 990 enrolled patients are presented in Table 1. In total, 747 patients had no RV dilatation, and 243 patients had RV dilatation at baseline.

**Table 1:** Baseline patient characteristics of the study population

	No pre-existing RV dilatation *N* = 747	Pre-existing RV dilatation *N* = 243	*P* value
Age (years)	70.4 ± 12.5	70.4 ± 10.9	0.95
Female sex	285 (38.2%)	92 (37.9%)	0.94
BMI (kg/m^2^)	26.5 ± 4.7	26.7 ± 4.7	0.67
BSA (m^2^)	1.89 ± 0.23	1.89 ± 0.23	0.63
Single-chamber pacemaker	158 (23.1%)	82 (40.8%)	**< 0.001**
RV pacing threshold (V)	0.71 ± 0.61	0.75 ± 0.63	0.80
Coronary artery disease	420 (56.2%)	147 (60.5%)	0.24
Heart failure	375 (50.2%)	145 (59.7%)	**0.01**
Diabetes	191 (25.6%)	68 (28.0%)	0.46
Myocarditis	3 (0.4%)	1 (0.4%)	0.98
Any atherosclerosis	498 (66.7%)	161 (66.3%)	0.91
Previous stroke or TIA	94 (12.6%)	32 (13.2%)	0.81
Previous CABG	55 (7.4%)	38 (15.6%)	**< 0.001**
Atrial fibrillation	385 (51.5%)	169 (69.5%)	**< 0.001**
Chronic kidney disease	183 (24.5%)	95 (39.1%)	**< 0.001**
Endocarditis	58 (7.8%)	22 (9.1%)	0.52

Values are mean ± SD, *N* (%), or median (interquartile range), unless otherwise indicated. *P* values < 0.05 in bold

*BMI* body mass index, *BSA* body surface area, *CABG* coronary artery bypass grafting, *RV* right ventricle, *TIA* transient ischemic attack, *V* volt

Patients with RV dilatation had more atrial fibrillation and higher prevalence of indications for single-chamber pacemaker. Patients with RV dilatation had more CABG, but rates of coronary artery disease showed no difference. Heart failure was more common in patients with RV dilatation. Patients with RV dilatation exhibited a higher prevalence of chronic kidney disease.

Echocardiographic parameters are presented in Table 2. Patients with RV dilatation had more severe degrees of tricuspid regurgitation both pre- and post-intervention, and pre-interventional TR-*V*_max_ and sPAP were higher. Regarding left heart disease, the group with RV dilatation had worse LVF, higher rates of mitral regurgitation, and larger dimensions of the left ventricle and the left atrium.

**Table 2:** Echocardiographic patient profile

	No pre-existing RV dilatation *N* = 747	Pre-existing RV dilatation *N* = 243	*P* value
*Pre-pacemaker*			
No or mild TR	579 (77.5%)	148 (60.9%)	**< 0.001**
Moderate TR	131 (17.5%)	60 (24.7%)	
Severe TR	37 (5.0%)	35 (14.4%)	
*Post-pacemaker*			
No or mild TR	476 (63.7%)	75 (30.9%)	**< 0.001**
Moderate TR	216 (28.9%)	85 (35.0%)	
Severe TR	55 (7.4%)	83 (34.2%)	
*Pre-pacemaker*			
Left ventricular function			
Normal	431 (57.8%)	97 (39.9%)	**< 0.001**
Mild reduction	121 (16.2%)	44 (18.1%)	
Moderate reduction	100 (13.4%)	35 (14.4%)	
Severe reduction	94 (12.6%)	67 (27.6%)	
*Mitral regurgitation*			
No or mild	343 (53.3%)	61 (32.3%)	**< 0.001**
Moderate	254 (39.4%)	83 (43.9%)	
Severe	47 (7.3%)	45 (23.8%)	
sPAP (mmHg)	44.7 ± 14.2	58.2 ± 17.6	**< 0.001**
LVEDD indexed (mm/m^2^)	25.4 ± 4.4	27.2 ± 5.4	**< 0.001**
LA (mm)	61 ± 10	70 ± 11	**< 0.001**
RVEDD (mm)	33 ± 4	43 ± 6	**< 0.001**
*Right ventricular function*			
Normal	598 (85.2%)	86 (39.4%)	**< 0.001**
Mild reduction	76 (10.8%)	64 (29.4%)	
Moderate reduction	21 (3.0%)	48 (22.0%)	
Severe reduction	7 (1.0%)	20 (9.2%)	
RA (mm)	59 ± 10	72 ± 45	**< 0.001**
TR-*V*_max_ (m/s)	2.93 ± 0.64	3.09 ± 0.65	**0.004**

Values are mean ± SD, *N* (%), or median (interquartile range), unless otherwise indicated. *P* values < 0.05 in bold

*LA* left atrium, *LVEDD* left ventricular end-diastolic diameter, *RA* right atrium, *RV* right ventricle, *RVEDD* right ventricular end-diastolic diameter, *sPAP* systolic pulmonary artery pressure, *TR* tricuspid regurgitation, *TR-V*_*max*_ peak tricuspid regurgitation velocity

### Progression of tricuspid regurgitation

**Fig. 1:**
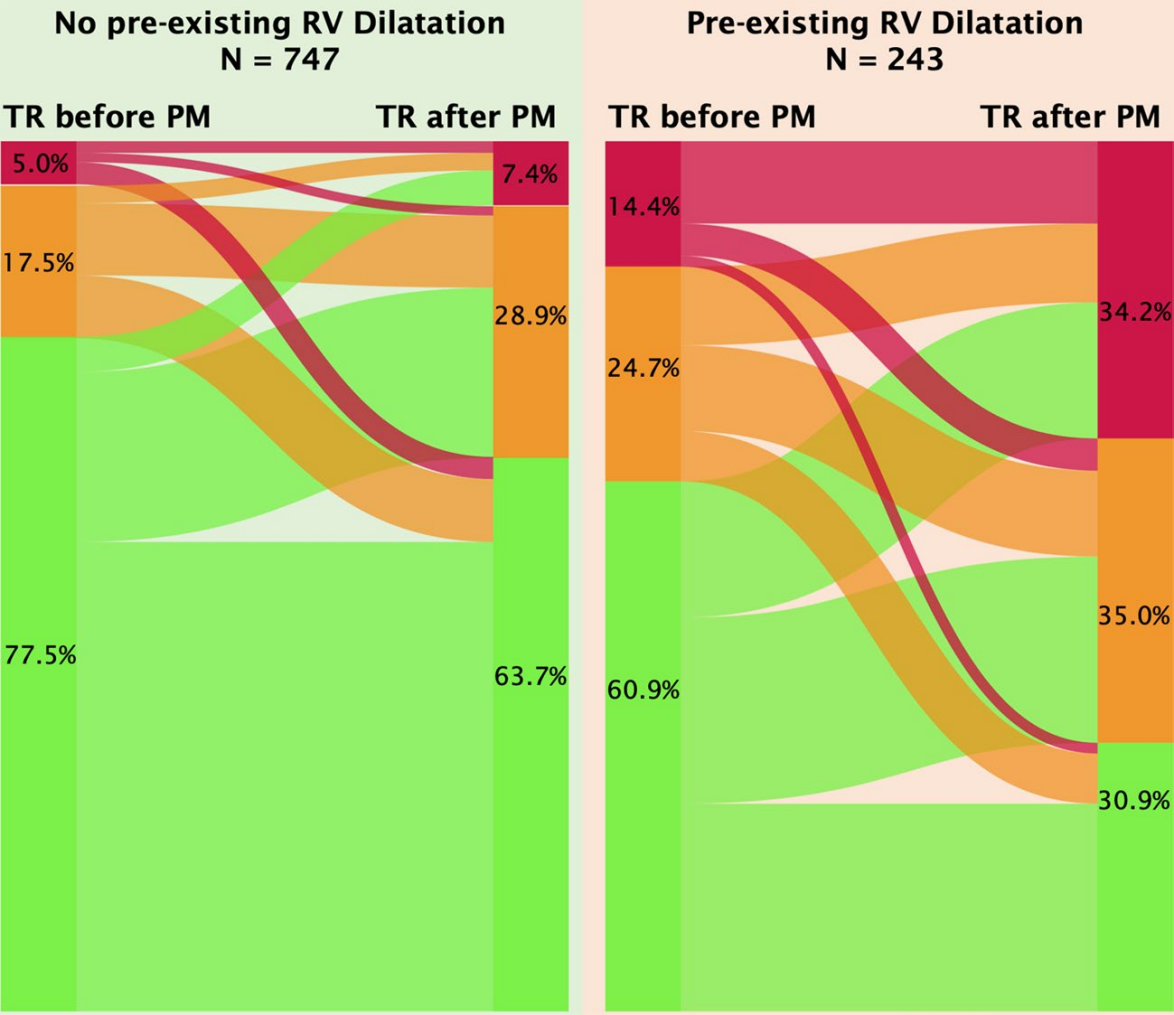
Tricuspid regurgitation before and after implantation of pacemaker. Sankey chart of grade of pre-existing TR with visualized flow to post-interventional TR after pacemaker implantation. Green: no/mild TR, orange: moderate TR, red: severe TR. Left: patients without RV dilatation. Right: patients with RV dilatation. Grades of TR are displayed in %. *PM* pacemaker, *TR* tricuspid regurgitation

**Fig. 2:**
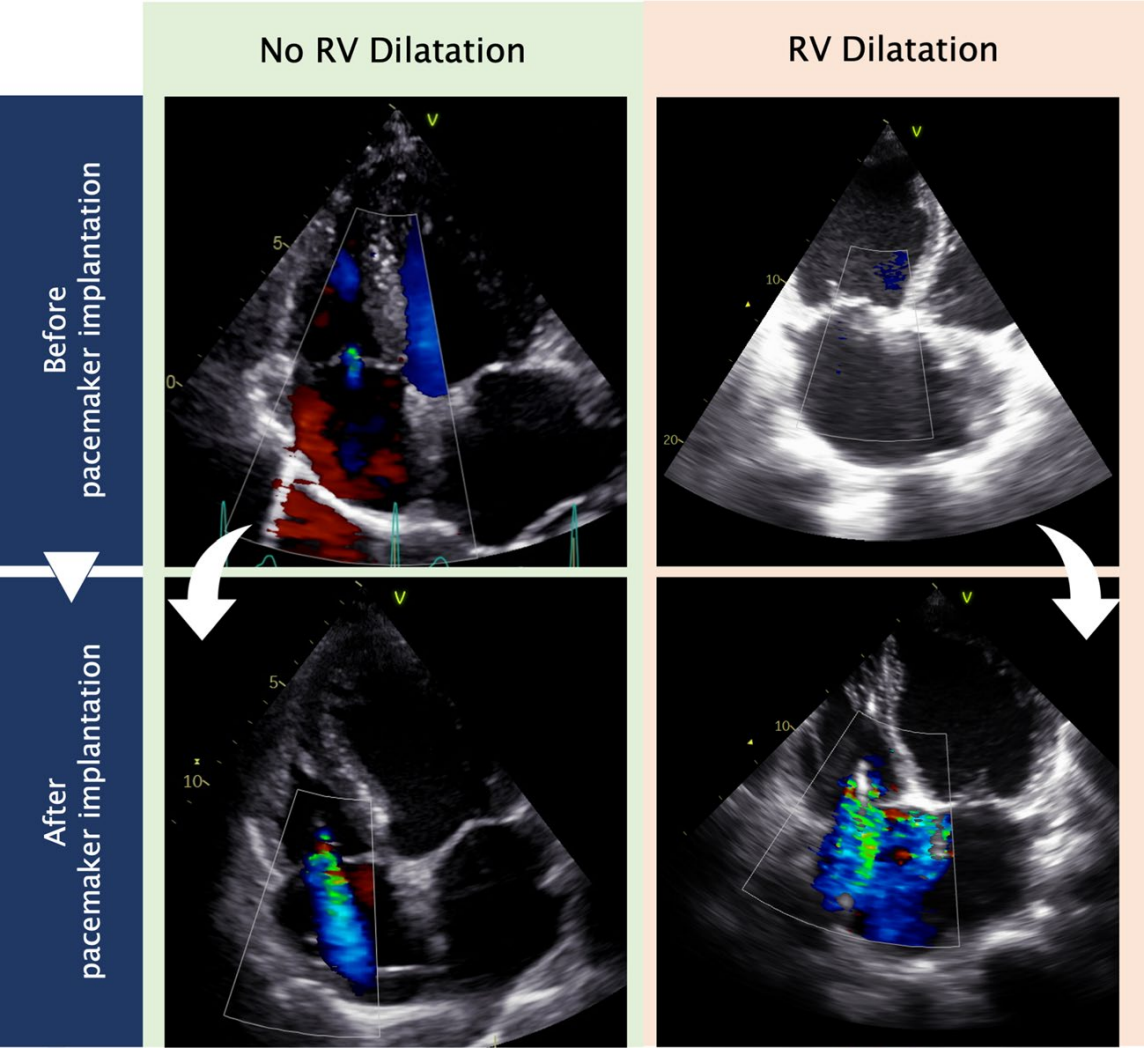
Echocardiography images evaluating tricuspid regurgitation before and after pacemaker implantation. Representative images from transthoracic echocardiography (4-chamber view) evaluating tricuspid regurgitation before and after pacemaker implantation in a patient without right ventricular dilatation (left column) and in a patient with right ventricular dilatation (right column)

In total, 303 out of 990 patients (30.6%) had progression of TR after pacemaker lead implantation, as shown in Fig. 1 and in Supplemental Fig. 1. Representative echocardiography studies evaluating TR before and after pacemaker implantation are displayed in Fig. 2. Pacemaker lead-associated progression of TR occurred in 112 out of 243 patients with RV dilatation (46.1%) compared to 191 out of 747 patients without RV dilatation (25.6%; *P* < 0.001). The odds ratio (univariate) for progression of TR was increased if RV dilatation was prevalent (OR 2.49; 95% CI 1.84–3.36; *P* < 0.001, compared to normal RV dimensions).

Using an ordinal regression model, the probability to suffer from severe TR after pacemaker implantation in patients with no or mild pre-existing TR and without RV dilatation was 6.3% (95% CI 4.7–8.3%), compared to 21.5% (95% CI 21.4–21.6%) in patients with RV dilatation. The probability to suffer from severe TR after pacemaker implantation in patients with pre-existing moderate TR and without RV dilatation was 14.8% (95% CI 11.0–19.7%), compared to 41.6% (95% CI 40.3–42.8%) in patients with RV dilatation.

Improvement of TR of at least one grade after pacemaker implantation occurred in 10.7% (*N* = 26) of patients with RV dilatation and in 10.8% (*N* = 81) of patients without RV dilatation (*P* = 0.95).

### Risk factors for progression of TR

Risk factors for progression of TR are shown in Table 3. After adjustment for patient baseline characteristics and echocardiographic parameters, the following characteristics were independently associated with progression of TR: RV dilatation (OR 2.04; 95% CI 1.27–3.29; *P* = 0.003), moderate pre-existing TR (OR 4.30; 95% CI 2.51–7.38; *P* < 0.001), female sex (OR 1.68; 95% CI 1.16–2.43; *P* = 0.006), single RV lead (OR 1.67; 95% CI 1.09–2.56; *P* = 0.018), moderate or severe mitral regurgitation (OR 2.08; 95% CI 1.42–3.05; *P* < 0.001), and an enlarged left atrium of ≥ 70 mm (OR 1.98; 95% CI 1.07–3.67; *P* = 0.03).

**Table 3:** Predictors for progression of tricuspid regurgitation

	Univariate		Multivariate	
	OR (95% CI)	*P* value	OR (95% CI)	*P* value
RV Dilatation	2.49 (1.84–3.36)	< 0.001	2.04 (1.27–3.29)	**0.003**
RVF Reduction	1.66 (1.22–2.27)	0.001	0.97 (0.60–1.56)	0.890
Pre-existing TR (moderate)	2.08 (1.41–3.06)	< 0.001	4.30 (2.51–7.38)	**< 0.001**
Age ≥ 80	1.11 (0.79–1.54)	0.551		
Female sex	1.39 (1.06–1.83)	0.018	1.68 (1.16–2.43)	**0.006**
Single RV lead	1.62 (1.18–2.21)	0.003	1.67 (1.09–2.56)	**0.018**
Coronary artery disease	1.36 (1.03–1.79)	0.031	0.98 (0.49–1.94)	0.950
Heart failure	1.68 (1.28–2.21)	< 0.001	1.39 (0.88–2.19)	0.159
Diabetes	0.99 (0.73–1.35)	0.966		
Any atherosclerosis	1.28 (0.96–1.71)	0.099	1.13 (0.56–2.28)	0.726
Atrial fibrillation	1.58 (1.20–2.09)	0.001	1.15 (0.78–1.69)	0.474
Chronic kidney disease	1.35 (1.003–1.81)	0.048	1.04 (0.71–1.54)	0.831
Endocarditis	1.48 (0.93–2.38)	0.101		
Mitral regurgitation (moderate or severe)	2.22 (1.63–3.02)	< 0.001	2.08 (1.42–3.05)	**< 0.001**
sPAP ≥ 56 mmHg	2.16 (1.47–3.15)	< 0.001	1.18 (0.69–2.05)	0.546
LVEDD ≥ 25 mm/m^2^	1.33 (1.01–1.75)	0.04	1.09 (0.75–1.57)	0.663
LA ≥ 70 mm	2.30 (1.69–3.13)	< 0.001	1.98 (1.07–3.67)	**0.030**
RA ≥ 70 mm	2.03 (1.47–2.82)	< 0.001	0.75 (0.38–1.46)	0.396

List of parameters included in the uni- and multivariate regression. All echocardiographic parameters were obtained from the echocardiographic study before pacemaker implantation. Parameters were included in the multivariate regression model with a *P* value < 0.10 in the univariate regression

*CI* confidence interval, *LA* left atrium, *LVEDD* left ventricular end-diastolic diameter, *OR* odds ratio, *RA* right atrium, *RV* right ventricle, *RVF* right ventricular function, *sPAP* systolic pulmonary artery pressure, *TR* tricuspid regurgitation

### Mortality

**Fig. 3:**
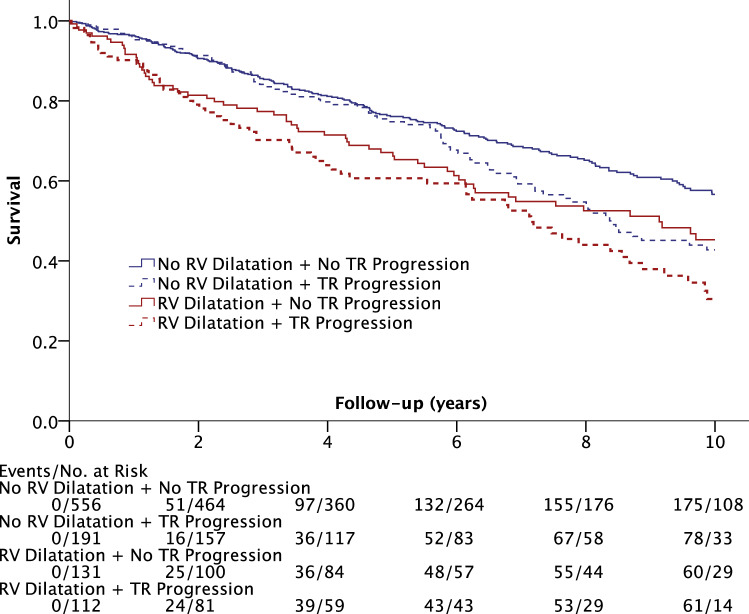
Survival of patients with/without right ventricular dilatation and with/without pacemaker lead-associated progression of tricuspid regurgitation. Kaplan–Meier plot of included patients with a 10-year follow-up. No. of events and patients at risk (No. at risk) are given in total numbers at year 0, 2, 4, 6, 8, and 10. *RV* right ventricle, *TR* tricuspid regurgitation

During the follow-up, 5569 person-years were analyzed, and the median follow-up time was 5.6 years (IQR 2.8–8.9 years). Completed 10-year follow-up was available for 562 of 990 patients, with a mortality rate of 62.2% of these 562 patients. Figure 3 indicates survival shown by Kaplan–Meier plot.

In patients without RV dilatation, lead-associated progression of TR had a higher mortality rate compared to non-progressors (HR 1.35; 95% CI 1.03–1.76; *P* = 0.028). The 10-year survival rate was 68.5% vs. 59.2% (non-progressors vs. progressors without RV dilatation). Patients with RV dilatation had no statistically significant differences in survival if lead-associated progression of TR was present (HR 1.33; 95% CI 0.93–1.89; *P* = 0.12). The 10-year survival rate was 54.2% vs. 45.5% (non-progressors vs. progressors with RV dilatation).

Table 4 shows the results of univariate and multivariate COX regression. After adjustment for relevant comorbidities and echocardiographic parameters, independent factors for decreased survival were pacemaker lead-associated TR (HR 1.38; 95% CI 1.04–1.84; *P* = 0.028), moderate/severe mitral regurgitation (HR 1.34; 95% CI 1.02–1.77; *P* = 0.034), heart failure (HR 1.75; 95% CI 1.31–2.33; *P* < 0.001), chronic kidney disease (HR 1.62; 95% CI 1.25–2.11; *P* = 0.001), and age ≥ 80 years (HR 2.84; 95% CI 2.17–3.71; *P* < 0.001).

**Table 4:** COX regression identifying predictors for mortality

	Univariate		Multivariate	
	HR (95% CI)	*P* value	HR (95% CI)	*P* value
RV Dilatation	1.58 (1.27–1.96)	< 0.001	0.95 (0.67–1.33)	0.747
RVF Reduction	1.58 (1.25–2.00)	< 0.001	1.14 (0.83–1.57)	0.413
Pre-existing TR (moderate)	1.34 (1.07–1.67)	0.01	1.30 (0.94–1.81)	0.116
Lead-associated TR progression	1.44 (1.17–1.78)	0.001	1.38 (1.04–1.84)	**0.028**
Age ≥ 80	2.78 (2.23–3.47)	< 0.001	2.84 (2.17–3.71)	**< 0.001**
Female sex	0.87 (0.71–1.08)	0.202		
Single RV lead	1.26 (0.99–1.60)	0.061	1.27 (0.96–1.69)	0.093
Coronary artery disease	1.10 (0.90–1.36)	0.35		
Heart failure	1.38 (1.12–1.69)	0.002	1.75 (1.31–2.33)	**< 0.001**
Diabetes	1.13 (0.90–1.41)	0.289		
Any atherosclerosis	0.99 (0.80–1.23)	0.944		
Atrial fibrillation	0.92 (0.75–1.12)	0.392		
Chronic kidney disease	1.55 (1.25–1.92)	< 0.001	1.62 (1.25–2.11)	**< 0.001**
Endocarditis	0.67 (0.44–1.02)	0.061	1.10 (0.61–1.99)	0.749
Mitral regurgitation (moderate or severe)	1.90 (1.51–2.37)	< 0.001	1.34 (1.02–1.77)	**0.034**
sPAP ≥ 56 mmHg	0.95 (0.68–1.32)	0.736		
LVEDD ≥ 25 mm/m2	1.59 (1.29–1.96)	< 0.001	1.29 (0.997–1.67)	0.053
LA ≥ 70 mm	1.21 (0.96–1.52)	0.108		
RA ≥ 70 mm	1.23 (0.97–1.56)	0.09	0.84 (0.60–1.18)	0.308
TR-*V*_max_ ≥ 3.5 m/s	1.55 (1.18–2.05)	0.002	0.99 (0.65–1.49)	0.945

List of parameters included in the uni- and multivariate COX regression. All echocardiographic parameters were obtained from the echocardiographic study before pacemaker implantation. Parameters were included in the multivariate COX regression with a *P* value < 0.10 in the univariate COX regression

*CI* confidence interval, *HR* hazard ratio, *LA* left atrium, *LVEDD* left ventricular end-diastolic diameter, *RA* right atrium, *RV* right ventricle, *RVF* right ventricular function, *sPAP* systolic pulmonary artery pressure, *TR* tricuspid regurgitation

### Lead replacement rate

Replacement of at least one pacemaker lead was necessary in 94 out of 990 patients (9.5%). Patients with RV dilatation had a higher risk for lead replacement (12.8% of patients with RV dilatation vs. 8.4% of patients without RV dilatation, OR 1.59; 95% CI 1.01–2.51; *P* = 0.047). Neither the grade of TR before or after PM implantation nor the progression of TR was associated with higher risks for lead replacement.

## Discussion

This is the first study investigating the link between RV dimensions and the risk of pacemaker lead-associated progression of TR. The primary findings are: (1) Patients with prior RV dilatation had an increased probability of progression of TR. (2) Beside RV dimensions, independent predictors for pacemaker lead-associated TR were pre-existing TR (at least moderate), female sex, single RV lead, and moderate/severe mitral regurgitation. (3) Independent survival predictors included lead-associated TR, mitral regurgitation, heart failure, chronic kidney disease, and age ≥ 80 years.

Previous studies have reported a high incidence of TR progression after CIED implantation. In our study, 30.6% of patients exhibited progression of TR after pacemaker implantation, which is comparable to 38% reported by Höke et al. [16], but significantly higher than the 21.2% found by Kim et al. [26], 18.3% found by Klutstein et al. [27], or 13% found by Seo et al. [19].

The main reason for the discrepancies between these studies lies in differing definitions of significant TR and of progression of TR. Our study included all grades of TR, had liberal definitions of progression of TR, and is most comparable to the design and definitions of Höke et al.: All grades of TR were included, and the echocardiography were performed from 1 to 1.5 years after CIED implantation, which is comparable to our study [16]. Compared to Höke et al., our analysis included more patients with longer follow-up and excluded patients with ICD or CRT, resulting in a more homogenous patient collective with focus on the properties of the right ventricle.

Seo et al. reported a rate of TR progression of only 13%, which is the lowest value reported in all comparable studies [19]. Importantly, they excluded all patients with moderate or severe TR before CIED implantation, and defined worsened TR by progression to moderate or severe TR after CIED implantation. Our study included the whole spectrum of TR, which explains the discrepancy.

Our study cohort exclusively contains patients with pacemakers, as patients with CRTs or ICDs were excluded. Inclusion of CRT would skew the results because of modified post-interventional rates of mitral regurgitation due to enhanced LVF. Furthermore, biventricular pacing may modify the risk of secondary CIED-related TR. The increased thickness and stiffness of ICD leads have been reported to cause higher rates of lead-associated TR, which is the reason for exclusion from our cohort [26]. Additionally, patients with CRTs or ICDs generally suffer from lower LVF and increased mortality compared to pacemaker patients [28].

Atrial fibrillation was found to represent a risk factor for CIED-associated progression of TR [17, 29]. In our univariate regression for progression of TR, atrial fibrillation and single ventricular pacing lead was found to be significantly associated with progression of TR. In the multivariate analysis, only single ventricular pacing lead remained as a risk factor. This could be still due to atrial fibrillation, as atrial fibrillation is the major indication for implantation of a pacemaker with a single ventricular lead. Atrial fibrillation is a common driver for TR due to its dilatating effect on the right atrium, triggering coaptation defects of the tricuspid valve [30].

### Risk factors for progression of TR

Delling et al. reported no significant progression of TR after CIED implantation in 169 patients, but described independent risk factors for TR in patients with CIED, which included age, body mass index, heart rate, right ventricular dilatation, sPAP ≥ 37 mmHg, history of mitral valve repair or replacement, and severe mitral regurgitation. Although the number of patients with echocardiography both before and after CIED implantation was low (*N* = 169), the study included 1245 patients with echocardiography only after CIED implantation. Analysis of that cohort revealed similar risk factors for TR with CIED, but lacked a direct comparison of TR before and after implantation. Additionally, analysis of mortality did not include right ventricular dilatation. In this context, our study provides substantial new insights regarding CIEDs, TR, and right ventricular properties, especially concerning RV dilatation.

### Survival

Association of CIED-induced progression of TR and mortality is well-known, and worsening of TR has been identified as an independent risk factor for mortality. Our observed hazard ratio of 1.38 for pacemaker lead-associated TR is comparable to the hazard ratio of 1.65 reported by Höke et al., and of 1.40 by Delling et al. The study by Seo et al. described a higher hazard ratio of 2.8 for TR progression after CIED implantation, but this is likely due to their stringent definition of TR progression.

### Improved TR after pacemaker implantation

Amelioration of TR after pacemaker implantation is occasionally seen. Whether TR improves after CIED mplantation depends on the hemodynamic severity of bradycardic disorders prior to CIED implantation [14, 27, 31]. After pacemaker implantation, the cardiac output may increase, the right ventricular pressure decreases, leading to improved TR in some patients.

### Clinical consequences

Better understanding of independent risk factors for pacemaker lead-associated TR and the associated increase in mortality might enable better risk stratification and therefore a more personalized treatment approach in patients with indication for pacemakers.

Possible options for patients with expected pacemaker lead-associated TR could include leadless pacing, HIS-bundle pacing or coronary sinus pacing. In a recent small-scale study, leadless pacing caused worsening of TR in 43% of 53 patients after a follow-up of 12 months [32]. This counterintuitive finding defies expectations but has yet to be replicated in larger trials. A possible mechanism might be functional TR caused by dyssynchrony.

If pacemaker lead-associated TR occurs, TTVI is an attractive option, as Taramasso et al. demonstrated feasibility and good clinical outcomes of TTVI in patients with TR and CIED [21]. However, some patients with primary severe CIED-induced TR may require surgery [33].

### Strengths

The main strengths of this study are the large sample size and the extended longitudinal follow-up with thorough survival analysis, and the definition of predictive factors for pacemaker lead-associated TR, facilitating personalized decision for TTVI.

## Limitations

The major limitation of this study is its retrospective nature. Residual confounding and selection bias might be an issue, highlighting the urgency for prospective trials. Patients without post-interventional echocardiography were not included in this trial. Some clinical characteristics were identified by ICD-10 codes, which are reliant on accurate recording by treating physicians. Although RV dilatation and TR are known drivers for further progression of TR independently of transtricuspid pacing leads, pacemaker leads represent a major independent risk factor for TR progression in a matched RV dilatation-cohort comparing patients with vs. without pacemakers. The only study from the literature which compares a large cohort of patients with and without pacemakers is cross-sectional hence can only describe a statistical instead of a functional association between transvenous pacemaker leads and both tricuspid regurgitation and mortality [15]. Patients with RV dilatation were in overall sicker than patients without RV dilatation. Our data on RV dilation and TR lack a control group of patients without pacemaker, which could be an acceptable method to adjust for contributing factors for progression of TR in patients (i.e., RV dilatation or pre-existing TR). Unfortunately, this study lack data from 3D-TTE, leading to the fact that the exact mechanism of progression of TR remained unknown and true severity of tricuspid regurgitation after pacemaker implantation could be underestimated. Reliable clinical 3D-TTE was not available in the majority of the included patients, as inclusion of patients started in the year 2000. Detailed echocardiographic data such as left ventricular ejection fraction (in %) or detailed measurements of TR (vena contracta, proximal isovelocity surface area) were not available, and interobserver variability has to be considered as a potential bias in studies based on echo data. Although data for lead replacements were analyzed, the reasons for the lead replacement were not available.

## Conclusion

Patients with prior RV dilatation suffered from higher rates of TR progression after pacemaker implantation, and patients with pacemaker lead-associated progression of TR exhibited impaired survival. Patients at risk for pacemaker lead-associated progression of TR could be treated with leadless pacemakers, and TTVI has to be further evaluated as a treatment strategy for patients with pacemaker lead-associated TR.
